# Topic Structure Affects Semantic Integration: Evidence from Event-Related Potentials

**DOI:** 10.1371/journal.pone.0079734

**Published:** 2013-12-02

**Authors:** Xiaohong Yang, Xuhai Chen, Shuang Chen, Xiaoying Xu, Yufang Yang

**Affiliations:** 1 Key Laboratory of Behavioral Science, Institute of Psychology, Chinese Academy of Sciences, Beijing, China; 2 School of Psychology, Shannxi Normal University, Xi'an, China; 3 School of Chinese Language and Literature, Beijing Normal University, Beijing, China; Harvard Medical School/Massachusetts General Hospital, United States of America

## Abstract

This study investigated whether semantic integration in discourse context could be influenced by topic structure using event-related brain potentials. Participants read discourses in which the last sentence contained a critical word that was either congruent or incongruent with the topic established in the first sentence. The intervening sentences between the first and the last sentence of the discourse either maintained or shifted the original topic. Results showed that incongruent words in topic-maintained discourses elicited an N400 effect that was broadly distributed over the scalp while those in topic-shifted discourses elicited an N400 effect that was lateralized to the right hemisphere and localized over central and posterior areas. Moreover, a late positivity effect was only elicited by incongruent words in topic-shifted discourses, but not in topic-maintained discourses. This suggests an important role for discourse structure in semantic integration, such that compared with topic-maintained discourses, the complexity of discourse structure in topic-shifted condition reduces the initial stage of semantic integration and enhances the later stage in which a mental representation is updated.

## Introduction

Recently, a heated topic on discourse comprehension has been how readers interpret upcoming information with discourse context. Although it seems clear that semantic integration is rapidly influenced by global discourse context, whether and how semantic integration interacts with global discourse structure is less clear-cut. In the current study, we used event-related potentials (ERPs) to examine whether and how semantic integration is affected by the processing of topic-shifted structure as compared to topic-maintained structure of a discourse.

A substantial body of studies has examined semantic integration in discourse context using eye movement and neurophysiological measures. Conflict between global discourse context and local information may result in longer reading times [1]–[3] or an N400 effect for inappropriate words as compared to appropriate words [1], [4]–[12]. The N400 is a central-parietal negativity, peaking around 400 ms post stimulus onset. It was first reported by Kutas & Hillyard [13] for semantic anomalies in sentence context. Generally, semantically incongruent words elicit larger N400s than semantically congruent words. This is referred to as an N400 effect (for a review see Kutas & Federmeier [14]). The general consensus is that the amplitude of the N400 is attenuated when the meaning of a word can be easily accessed and integrated into the preceding context.

In two ERP experiments, van Berkum et al [10] found evidence suggesting that unfolding words were related to the wider discourse rapidly. In their first experiment, participants listened to sentences such as “Jane told her brother that he was exceptionally *quick/slow*”, which were designed such that the critical words were always coherent in the local sentence context. However, the critical words were either semantically coherent or incoherent with respect to the wider discourse (e.g., “Jane told the brother that he was exceptionally slow” in a discourse context where he had in fact been very quick). Relative to coherent control words (e.g., quick), these discourse-dependent semantic anomalies elicited a large N400 effect that began at about 200 to 250 msec after word onset. The second experiment showed that when the same sentences were heard in isolation, the N400 effect disappeared. This suggests that the presence of the N400 effect was due to the mismatch between local information and discourse context.

Note that semantic anomalies may not always give rise to an N400 effect, but may produce a P600-effect instead. The P600 (also referred to as the late positivity) is a positive deflection starting at about 400 ms and reaches its maximum around 600 ms post stimulus onset. It is typically related to the process of syntactic revision and repair in sentence processing when the sentences are not syntactically well-formed (for a review see Gouvea, Phillips, Kazanina, & Poeppel [15]). However, this assumption has been challenged by recent evidence. In Nieuwland & Van Berkum [16], semantic anomalous words did not elicit an N400 effect, but a late positivity effect from about 500–600 ms post stimulus onset. This was unexpected since the materials were perfectly grammatical. The absence of an N400 effect suggests that subjects did not immediately notice the anomaly and could be interpreted as a short-lived semantic illusion. The late positivity effect, however, indicates that subjects were processing for comprehension and did ultimately detect the anomaly. Similar observations of a late positivity to semantic anomalies have also been reported in other studies (for a review see Brouwer, Fitz, & Hoeks [17]). To account for the late positivity for syntactically well-formed sentences, different explanations have been proposed. Besides syntactic repair, the late positivity has also been proposed to reflect a conflict monitoring process [18], a more general integration process including different kinds of information such as semantics, pragmatics, prosody and others [19]–[21], or the brain's natural elcetrophysiological reflection of updating a mental representation with new information [17], [22], [23].

Although the effect of context on local semantic processing has been repeatedly demonstrated in the literature, the effect of global discourse structure on semantic integration has not yet been studied. However, numerous studies in the discourse processing literature have long emphasized topic structure of a discourse as a global discourse factor that influences language processing [24]–[34]. According to the Structure Building framework, when encountering topic shifts, comprehenders suppress the current discourse representation and develop another discourse representation [24]. Studies on anaphoric inferences and ambiguity resolution have suggested that topic shifts create an interference effect such that a new structure is initiated and information related to the earliest topic becomes less available [25]–[28]. Furthermore, it has been found that topic shifts require extra processing time [29]–[32] and the introduction of a new topic has been found to induce an enhanced late positivity indexing discourse updating [33], [34].

These aforementioned studies have demonstrated that topic structure of a discourse may affect natural reading process. However, to date, nobody has examined how topic structure affects the semantic integration process in which local information is interpreted in the wider discourse context. Thus, in the present study, we investigated whether and how semantic integration in discourse context is influenced by topic structure.

To addresses these questions, we presented participants with discourses in which the last sentence contained a critical word that was either discourse-congruent or discourse-incongruent with respect to the topic established in the first sentence of the discourse. Meanwhile, their ERP responses were recorded. Discourse structure was manipulated in such a way that the topic of the discourse was either maintained or shifted throughout the passage. Four experimental conditions were compared: topic-shifted/congruent, topic-shifted/incongruent, topic-maintained/congruent, and topic-maintained/incongruent (see Table 1 for example stimuli).

**Table 1:** Example stimuli used in the current study.

**Lead-in sentence:**
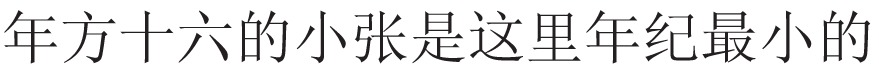
Zhang, who was only16 years old, was the youngest of all here.
**Topic-shifted intervening sentences:**
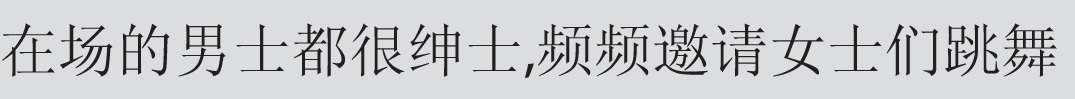
The gentlemen at the party were very courteous, and invited the ladies to dance.
**Topic-maintained intervening sentences:**
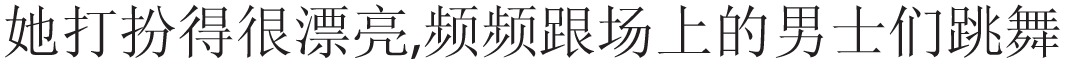
She dressed up gorgeously, and invited the gentlemen at the party to dance.
**Target sentence:**
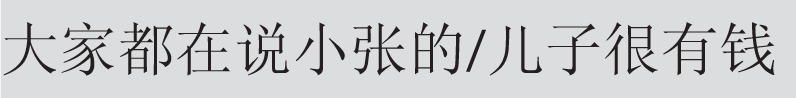
Everyone was saying that Zhang's **parents/son** were (was) very wealthy.

Note: In the experimental materials, either the topic-shifted or the topic-maintained intervening sentences were presented and either the congruent (parents) or incongruent target word (son) was presented. The English translations were presented below the Chinese sentences.

If, as suggested by previous studies, topic-maintained structure aids in keeping related concepts active in working memory while topic-shifted structure results in a interference effect that reduces the accessibility of relevant information [25]–[28], then discourse-dependent anomalies in topic-maintained condition should be detected with more ease and processed more elaborately while those in topic-shifted condition should be processed less elaborately. Consequently, a reduction of the N400 effect is predicted for incongruent words in the topic-shifted condition. Furthermore, based on previous studies showing that topic-shifted discourses require extra processing of discourse representation [29]–[32] and that the increased demand on discourse updating is reflected by the late positivity [22], [23], [33], [34], we expected incongruent words in topic-shifted discourses to elicit a late positivity effect, but a smaller or no late positivity effect in topic-maintained discourses.

## Methods

### Ethics Statement

All participants provided written informed consent in accordance with the Declaration of Helsinki. The ethics committee of the Institute of Psychology, Chinese Academy of Sciences approved this study, its participant-recruitment procedure and its methodology.

### Participants

Twenty-nine university students (15 women; mean age = 22.0 years; SD = 3.5) participated in the experiment for cash. All were native speakers of Chinese and were right-handed with normal or corrected-to-normal vision. Three of the participants were rejected from the final analysis due to EEG-artifacts, experimenter error or technical problems with recording. Thus, 26 participants remained for subsequent analysis.

### Materials

We constructed 160 sets of discourses. The first sentence was the lead-in sentence that established a topic. The last sentence contained a critical word which was either congruent or incongruent with the topic established in the first sentence. The intervening sentences between the first and the last sentence varied with discourse type (topic-shifted or topic-maintained). In the topic-shifted condition, the intervening sentences contained an overt shift in topic that departed away from the topic established in the first sentence. A topic shift was defined as a change in actor [26]. In the topic-maintained condition, however, the topic introduced in the first sentence was maintained throughout the intervening sentences. The incongruent and congruent words did not differ in average frequency (incongruent: *M* = 231.73, *SD* = 596.42; congruent: *M* = 170.63, *SD* = 312.13; *t*_(159)_ = 1.25, *p*>.1) based on information provided by Beijing Institute of Language [35]. The 160 discourses were counterbalanced and divided into four lists with each discourse set only presented once within each list. Each list contained 40 discourses per condition. To each list 80 filler discourses of various structures were added, half of which involved discourse anomalies.

Two pretests were conducted to assess the cloze probability and plausibility of the materials. In the first pretest, 17 participants were asked to write down the first word that came to mind to complete the discourses. The experimental stimuli were truncated before the critical word and randomly intermixed with the 80 filler discourses. Two lists were created in such a way that participants could only see the topic-maintained or the topic-shifted condition of the same item and there were an equal number of discourses from the two conditions. Results showed that no participant completed the sentences with the critical words that were used to create the incongruent condition. The mean cloze probability for the congruent words was 44.1% (SD = 7.8%) for the topic-shifted condition and 42.9% (SD = 7.6%) for the topic-maintained condition. A Paired-Samples t-test revealed no significant difference between the two structures (*t*_(16)_ = 0.49, *p*>.05).

In the second pretest, 20 participants were asked to rate the congruence of the discourses ranging from 1 (very implausible) to 5 (very plausible). The participants were assigned one of the four counterbalanced lists. Repeated-measures ANOVAs revealed a main effect of congruence (*F* (1,19) = 116.29, *p*<.001, η^2^_partial_ = .86; mean ± SD = 3.92±0.52; 1.77±0.57; 3.96±0.60; 1.84±0.55 for topic-shifted/congruent, topic-shifted/incongruent, topic-maintained/congruent, and topic-maintained/incongruent respectively), while no main effect of discourse structure or interaction was found (*Fs*<1.4).

### Procedure

Participants were tested individually in a sound-attenuating shielded chamber. They were seated in a comfortable chair approximately 60 cm in front of a monitor and were instructed to read the discourses for comprehension. The discourses were presented at the center of the computer screen. Trials began with a fixation cross which remained on the screen for 1000 ms. Then the first three sentences of the discourses appeared on the screen sentence by sentence. Participants were told to press a button when they finished reading the sentences. The last sentences were presented one word at a time. Each word was presented for 400 ms, followed by a blank screen for another 200 ms. The participants were free to move or blink during the presentation of the sentences but were instructed not to move or blink during the presentation of the words on the computer screen. After 1/4 of the trials they were asked to respond to a true/false comprehension question by pressing one of the two appropriately marked keys. Half of the questions required a “True” response and half a “False” response. After a short practice of 12 trials, the materials were presented in four blocks of approximately 10 min, separated by brief resting periods.

### EEG Recording and analysis

EEG was recorded with 64 Ag/gCl electrodes mounted in an elastic cap according to the international 10–20 system. Recording and digitization were carried out with a SynAmps amplifier 1 and Scan 4.3 Acquisition Software (Neuroscan Labs, TEXAS, USA). The EEG was sampled at 500 Hz with an amplifier bandpass of 0.01–100 Hz. Vertical EOG was recorded by electrodes placed above and below the left eye. The horizontal EOG was recorded via electrodes placed at the outer canthus of each eye. EEG data were referenced online to the left mastoid and re-referenced offline to the algebraic average of both mastoids. All electrode impedances were kept below 5 kΩ.

EEG recordings were processed offline and ERPs were computed using the Edit program in Scan 4.3 (Neuroscan Labs, TEXAS, USA). EEG and EOG records were screened for eye movements, electrode drifting, and excessive EEG amplitude exceeding ±75 µV. Contaminated trials were discarded (16.7% overall). The analysis epoch started 200 ms prior to the onset of the critical word, and lasted for 1000 ms after the onset of the critical words. Averages were aligned to a 200-ms baseline preceding the critical word.

**Figure 1:**
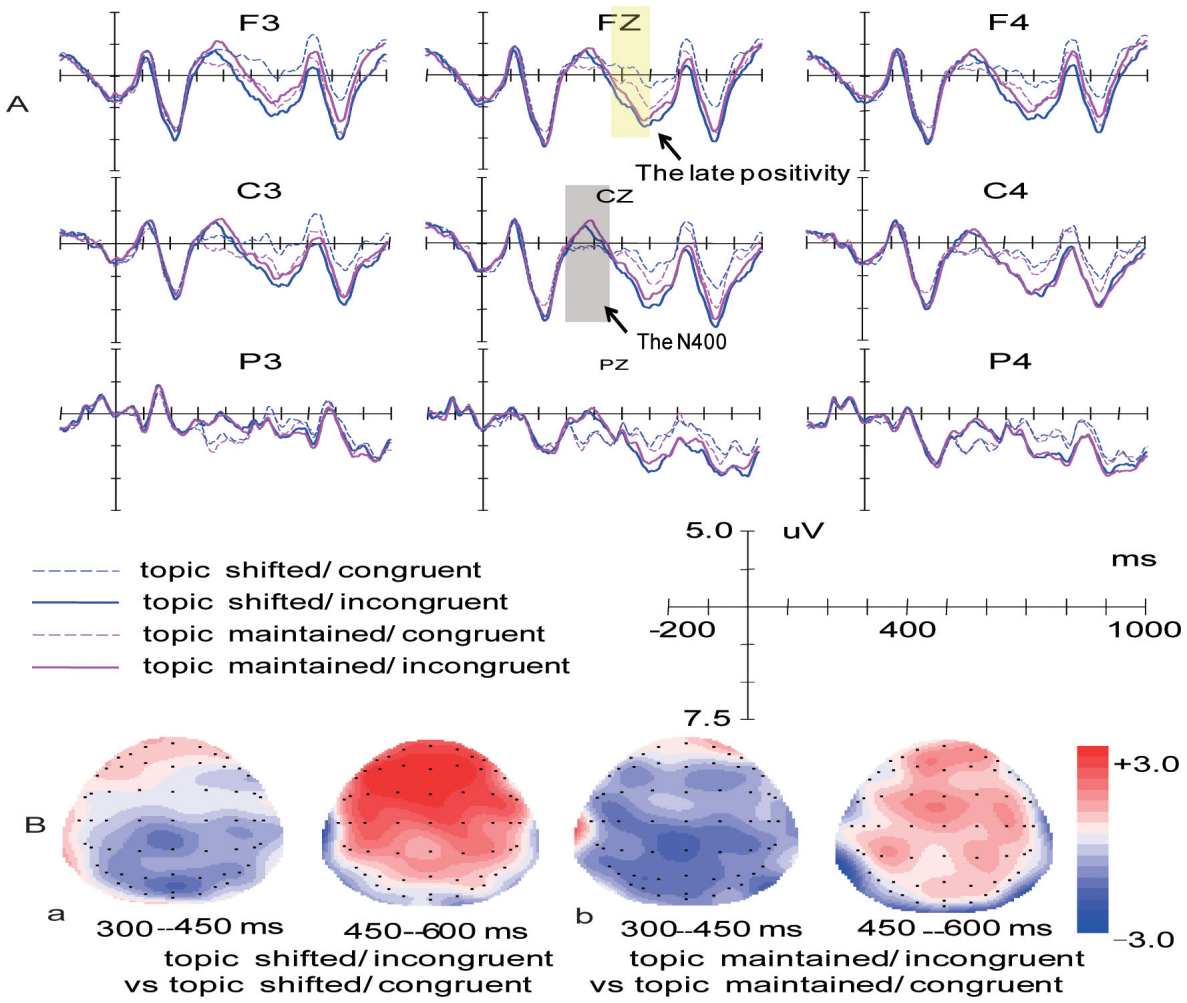
Grand-average ERP waveforms of the current Experiment. (A) Grand average waveforms elicited by the four conditions at selected electrode sites. Waveforms are time-locked to the onset of the critical words and negative amplitude is plotted up. (B) Topographies of the difference wave formed by subtracting ERPs to the topic shifted/congruent from topic shifted/incongruent (a), and ERPs to the topic maintained/congruent from topic maintained/incongruent (b) in selected time periods.

**Figure 2:**
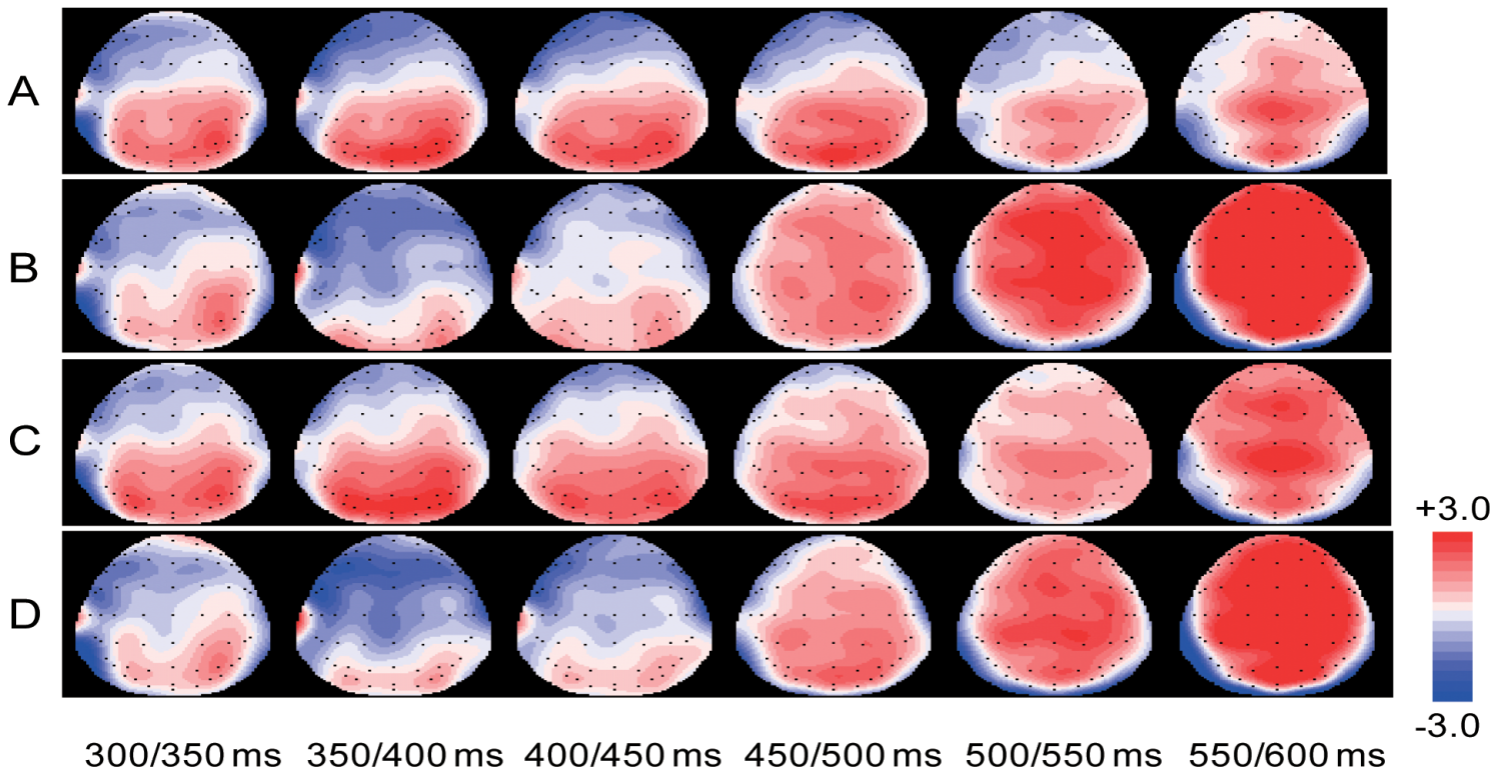
Topographical plot per condition for each 50–600 ms. (A): topic-shifted congruent. (B) topic-shifted incongruent. (C) topic-maintained congruent. (D) topic-maintained incongruent.

Average waveforms were computed across all trials per condition for the 26 participants. Visual inspection of Fig. 1 and 2 suggests that at least two distinct components of ERPs were modulated by the experimental variables: the N400 emerging around 300 ms and the late positivity emerging around 450 ms. Therefore, based on visual inspection of Fig. 1 and 2, we decided to analyze the N400 effect in a early time window from 300 to 450 ms. In our procedure, at 600 ms post target presentation another stimulus was presented. Therefore, to avoid the confound of the presentation of post target stimulus, we decided to analyze the late positive effect in a time window following the N400 effect but preceding the onset of the post target stimulus (450–600 ms).

Analyses of variance (ANOVAs) were conducted with topic structure (topic-shifted vs. topic-maintained) and congruence (congruent vs. incongruent) as independent variables. In order to cover distributional differences in both the anterior–posterior as well as in the left–right dimensions, two topographical factors were also included in the ANOVAs. The first topographical factor was ant–pos, which had six levels: frontal, frontal-central, central, central-parietal, parietal, and occipital ([36] [37]). The second topographical factor was laterality, which had three levels: left, medial, and right ([37] [38]). Thus, 18 regions of interests (ROI) were computed out of 51 electrodes, each containing 3 or 2 electrodes: left frontal (F3, F5, F7), left frontal-central (FC3, FC5, FT7), left central (C3, C5), left central-parietal (CP3, CP5, TP7), left parietal (P3, P5, P7), left occipital (P03, P07, O1), medial frontal (F1, FZ, F2), medial frontal-central (FC1, FCZ, FC2), medial central (C1, CZ, C2), medial central-parietal (CP1, CPZ, CP2), medial parietal (P1, PZ, P2), medial occipital (POZ, OZ), right frontal (F4, F6, F8), right frontal-central (FC4, FC6, FT8), right central (C4, C6), right central-parietal (CP4, CP6, TP8), right parietal (P4, P6, P8), and right occipital (P04, P08, O2). There is a fundamental incompatibility between the additive ANOVAs model and the multiplicative effect on ERP voltages produced by differences in source strength. For example, a 2-fold increase in source strength produces a corresponding 2-fold increase in the voltage at each location instead of adding a constant voltage to each location as assumed by the ANOVA model. Consequently, significant interactions involving electrode location can be obtained between scalp distributions with identical shapes generated by the same source. Therefore, such interactions cannot be used as unambiguous indications of shape differences between distributions and hence of differences in source configuration. This ambiguity can be circumvented by suitable scaling of the data before an ANOVA is performed (McCarthy and Wood [39]). Consequently, we normalized data following the procedure proposed by McCarthy and Wood [39]. In all those cases where interactions with site or hemisphere are reported, the normalized data are presented. When the degree of freedom in the numerator was larger than one, the Greenhouse–Geisser correction was applied. All statistical analyses were performed using SPSS16.0.

## Results

As shown in Fig. 1, in the 300–450 ms interval, discourse-dependent anomalous words elicited an N400 effect, which was largest over central-posterior electrodes. The N400 effect was followed by a frontal-central late positivity effect emerging around 450 ms. Closer inspection of the N400 time window reveals that over the right hemisphere, there is a clear N400 effect in the incongruent conditions which seems to be identical in both discourse conditions. However, over the left hemisphere, the N400 effect seems to be reduced in the topic-shifted condition. Furthermore, as is apparent from Fig. 1, the late positivity effects elicited by the topic-shifted and topic-maintained discourses show difference patterns at frontal and central areas. Whereas incongruent words in the topic-shifted condition generated a late positivity effect, those in the topic-maintained condition elicited a much reduced late positivity effect.

### Behavioral measures

Performance accuracy was above 85% for all participants for all experimental conditions. Repeated-measures ANOVA revealed neither the main effect of topic structure or congruence, nor the interaction between the two factors (*Fs<1*, *ps>.1*). This indicates that subjects were reading the sentences attentively and they paid attention equally well to the topic-shifted and the topic-maintained discourses.

### N400 Window (300–450 ms)

The overall analysis of N400 amplitude showed a main effect of congruence (as shown in Table 2). Mean amplitude was more negative going for the incongruent words than the congruent words, indicating that an N400 effect was obtained. The congruence×ant-pos interaction was significant. Further simple effect analysis indicated that the N400 effect was stronger over central and posterior electrodes [frontal: F (1, 25) = 0.43, *p*>.1, *η* ^2^_partial_ = 0.01; frontal-central: F (1, 25) = 1.91, *p*>.1, *η* ^2^_partial_ = 0.07; central: F (1, 25) = 8.35, *p*<.01, *η* ^2^_partial_ = 0.25; central-parietal: F (1, 25) = 3.79, *p* = .06, *η* ^2^_partial_ = 0.13; parietal: F (1, 25) = 24.46, *p*<.001, *η* ^2^_partial_ = 0.50; and occipital: F (1, 25) = 34.64, *p*<.001, *η* ^2^_partial_ = 0.58]. More importantly, there was a marginally significant three-way interaction between topic structure, congruence, and laterality.

**Table 2:** Results of the ANOVAs in the 300–450 ms and 450–600 ms Latency Range.

	300–450 ms				450–600 ms		
Source	df	*F*	*p*	*η* ^2^_partial_	*F*	*p*	*η* ^2^_partial_
C	1, 25	9.60	<.01	0.28	5.92	<.05	0.19
T	1, 25	0.10	0.75	0.00	0.60	0.46	0.02
C×A	5,125	5.64	<.01	0.18	9.59	<.001	0.28
C×L	2,50	0.25	0.71	0.01	4.82	<.05	0.16
C×A×L	10, 250	0.89	0.41	0.03	1.00	0.39	0.04
C×T	1, 25	2.49	0.12	0.09	3.31	0.08	0.12
C×T×L	2, 50	2.75	0.07	0.10	2.13	0.14	0.08
C×T×A	5, 125	0.88	0.43	0.03	2.43	0.08	0.08
C×T×A×L	10, 250	1.22	0.31	0.05	1.42	0.25	0.05

Note: The ANOVAs were based on the mean amplitude in 300–450 ms and 450–600 ms latency ranges. They included the following experimental variables: Topic structure (T, topic-shifted vs. topic-maintained), Congruence (C, congruent vs. incongruent), ant–pos (A), and laterality (L).

As the aim of the present study was to examine the impact of topic structure on semantic integration, we broke down the three-way interaction by the variable topic structure and calculated separate follow-up analyses within the topic-shifted (congruent vs. incongruent) and the topic-maintained (congruent vs. incongruent) conditions testing the variable congruence in left, medial, and right hemisphere. For the topic-shifted conditions, ANOVA with congruence and laterality as two within-participant factors revealed a marginally significant main effect of congruence [F (1, 25) = 4.16, *p* = .052, *η* ^2^_partial_ = 0.14] and a significant congruence×laterality interaction [F (2, 50) = 3.44, *p*<.05, *η* ^2^_partial_ = 0.12]. Further simple effect analysis for the two-way interaction revealed that an N400 effect was only present at the right hemisphere [left: F (1, 25) = 0.94, *p*>.1, *η* ^2^_partial_ = 0.03; medial: F (1, 25) = 2.87, *p*>.1, *η* ^2^_partial_ = 0.10; and right: F (1, 25) = 7.61, *p*<.05, *η* ^2^_partial_ = 0.23]. For the topic-maintained conditions, ANOVA with congruence and laterality as two within-participant factors revealed only a significant main effect of congruence [F (1, 25) = 9.39, *p*<.01, *η* ^2^_partial_ = 0.27]. No interaction of congruence with laterality was observed (*Fs*<1.5, *ps*>.1). These findings indicated that the N400 effect elicited by topic-maintained condition was broadly distributed over the scalp.

Although the overall analysis did not indicate significant anterior to posterior differences in the scalp distribution of the N400 effect elicited by the topic-shifted and topic-maintained conditions, inspection of Fig. 1 suggests that the N400 effects elicited by the topic-shifted and topic-maintained discourses differed in the frontal locations, but were identical over the central and posterior areas. To test this observation more formally, we conducted additional ANOVAs for the topic-shifted and topic-maintained conditions testing the variable congruence over the six ant–pos levels. For the topic-shifted condition, an ANOVA with congruence and ant–pos as two within-participant factors revealed a marginally significant main effect of congruence [F (1, 25) = 4.16, *p* = .052, *η* ^2^_partial_ = 0.14] and a significant congruence×ant-pos interaction [F (5, 125) = 6.22, *p*<.01, *η* ^2^_partial_ = 0.20]. Further simple effect analysis showed that an N400 effect was obtained at central, central-parietal, parietal, and occipital sites [frontal: F (1, 25) = 0.62, *p*>.1, *η* ^2^_partial_ = 0.02; frontal-central: F (1, 25) = 0.00, *p*>.1, *η* ^2^_partial_ = 0.00; central: F (1, 25) = 3.51, *p* = .07, *η* ^2^_partial_ = 0.12; central-parietal: F (1, 25) = 5.43, *p*<.05, *η* ^2^_partial_ = 0.18; parietal: F (1, 25) = 16.87, *p*<.001, *η* ^2^_partial_ = 0.40; and occipital: F (1, 25) = 14.87, *p*<.01, *η* ^2^_partial_ = 0.37]. For the topic-maintained condition, however, an ANOVA with congruence and ant–pos as two within-participant factors revealed only a significant main effect of congruence [F (1, 25) = 9.39, *p*<.01, *η* ^2^_partial_ = 0.27], indicating that the N400 effect elicited by the topic-maintained condition was distributed over the six ant–pos levels.

In summary, incongruent words elicited an N400 effect both in the topic-shifted condition and the topic-maintained condition. While the N400 effect in the topic-shifted condition was only found at the right hemisphere and localized to the central and posterior areas, the N400 effect elicited in the topic-maintained condition was found over the left, medial and right hemisphere and broadly distributed from frontal to posterior areas. This demonstrates that the N400 effect is broader in distribution in the topic-maintained condition than in the topic-shifted condition.

### Late positivity (450–600 ms)

The overall analysis for the late positivity revealed a significant main effect of congruence (as shown in Table 2). Mean amplitudes for incongruent words were more positive-going than those for congruent words. Furthermore, there were significant congruence×ant-pos and congruence×laterality interactions. Further simple effect analysis for the congruence×ant-pos interaction revealed that a late positivity was observed over the frontal [F (1, 25) = 16.83, *p*<.001, *η* ^2^_partial_ = 0.40], frontal-central [F (1, 25) = 17.22, *p*<.001, *η* ^2^_partial_ = 0.41], central [F (1, 25) = 4.56, *p*<.05, *η* ^2^_partial_ = 0.15], and central-parietal areas [F (1, 25) = 3.79, *p* = .06, *η* ^2^_partial_ = 0.13]. For the congruence×laterality interaction, further simple effect analysis revealed that a late positivity was observed over the left [F (1, 25) = 5.02, *p*<.05, *η* ^2^_partial_ = 0.17] and medial sites [F (1, 25) = 8.70, <.01, *η* ^2^_partial_ = 0.26].

More importantly, the overall analysis also revealed marginally significant topic structure×congruence and topic structure×congruence×ant-pos interactions. As with the analysis of the N400 effect, we resolved the three-way interaction by computing separate follow-up analyses within the topic-shifted and the topic-maintained conditions to examine the impact of topic structure on semantic integration. For the topic-shifted condition, an ANOVA with congruence and ant–pos as two within-participant factors revealed a significant congruence×ant-pos interaction [F (5, 125) = 6.83, *p*<.01, *η* ^2^_partial_ = 0.22]. Further simple effect analysis showed that a late positivity was obtained at frontal, frontal-central, and central-parietal areas [frontal: F (1, 25) = 22.91, *p*<.001, *η* ^2^_partial_ = 0.48; frontal-central: F (1, 25) = 22.20, *p*<.001, *η* ^2^_partial_ = 0.47; central: F (1, 25) = 1.52, *p*>.1, *η* ^2^_partial_ = 0.06; central-parietal: F (1, 25) = 6.31, *p*<.05, *η* ^2^_partial_ = 0.20; parietal: F (1, 25) = 0.59, *p*>.1, *η* ^2^_partial_ = 0.02; and occipital: F (1, 25) = 1.45, *p*>.1, *η* ^2^_partial_ = 0.06]. For the topic-maintained conditions, ANOVA with congruence and ant-pos as two within-participant factors revealed neither a significant main effect of congruence nor a significant interaction between congruence and ant-pos [Fs<.1.9, *ps*>.1].

In summary, a late positivity effect was elicited by incongruent words in the topic-shifted condition, but not by those in the topic-maintained condition.

## Discussion

This study aimed to investigate whether and how semantic integration is influenced by discourse structure. To examine this, we placed discourse-dependent anomalies in two kinds of discourse structure: topic-shifted and topic-maintained. Two important findings emerge from this study. Firstly, incongruent words in discourse context elicited an N400 effect followed by a late positivity effect, indicating that upcoming information was immediately related to the wider discourse context. Secondly and more importantly, we found that the N400 effect and the late positivity effect were modulated by discourse structure. The N400 effect is broader in distribution in the topic-maintained condition than in the topic-shifted condition. Moreover, the late positivity effect was only observed for incongruent words in the topic-shifted condition, but not for those in the topic-maintained condition. This suggests that shifts in discourse context reduce the initial stage of semantic integration and enhance the later stage in which a mental representation is updated.

### Effect of discourse-dependent anomaly

The result that incongruent words in discourse context elicited a classical N400 effect relative to congruent words is quite compatible with the current literature about this component. It has been suggested that the N400 is sensitive to the ease with which a word can be related to the wider discourse [14]. Note that the critical words in the current study were always congruent in the local sentence context and only became incongruent when interpreted with the preceding discourse context. Therefore, the observation that these discourse-dependent anomalies elicited a classical N400 effect confirmed that discourse context can immediately affect semantic integration, which corroborates nicely with previous findings [1]–[12].

In addition to the classical N400 effect, a frontalcentral late positivity effect was also elicited by incongruent words in the topic-shifted condition. The finding of a frontalcentral late positivity has been reported in the literature. Friederici, Hahne, & Saddy [40] distinguished two kinds of positivity: a centralparietal positivity indexing syntactic repair, and a frontalcentral positivity indexing sentence complexity. This was further supported by Kaan and colleagues [41], [42], who reported that a posteriorly distributed P600 was found to reflect syntactic repair and revision processes proper while a frontally distributed positivity was found to reflect ambiguity resolution and/or to an increase in discourse level complexity. In the current study, the late positivity effect was only observed in the topic-shifted condition, but not in the topic-maintained condition. This could be interpreted as providing further evidence that the frontalcentral positivity may be associated with increases in discourse complexity. The finding of a biphasic N400+late positivity is in line with the proposal put forward by Burkhardt [22], [23], in which two distinct levels of representation were introduced, one for the meaning of an utterance (reflected in the effect of N400), and one for the discourse representation (reflected in the effect of the late positivity).

### Effect of discourse structure on semantic integration

The primary aim of this study was to investigate whether topic structure influences semantic integration. The results obtained in the EPRs revealed that the incongruent words in the topic-maintained discourses elicited a broadly distributed N400 effect while those in topic-shifted discourses elicited a restrictedly distributed N400 effect followed by a late positivity effect. These results suggest that discourse structure modulates both the initial stage of semantic integration and the later stage of discourse updating.

Previous studies have suggested that a topic-maintained structure aids in keeping related concepts active in working memory while a shift in topic creates an interference effect that reduces the accessibility of relevant information [25]–[28]. Therefore, it is possible that in the current study, information related to the earlier topic was more available in topic-maintained discourses. Therefore, semantic integration was more fully executed, which resulted in a broadly distributed N400 effect. On the contrary, since topic-shifted structure created an interference effect such that information related to the earlier topic was less available, therefore, semantic information was less elaborately processed, as reflected by the restrictedly distributed N400 effect. This result adds to the literature by showing that discourse structure has an immediate influence on semantic integration.

Compared with topic-maintained discourses, a late positivity effect was found in response to incongruent words in topic-shifted discourses, but not in topic-maintained discourse. This may be due to the fact that the construction of a coherent representation involves more work in topic-shifted discourses than in topic-maintained discourses [24]. Previous studies have reported extra processing time for sentences that introduce a new subtopic in the text [29]–[32]. In the current study, the intervening sentences in the topic-shifted discourses always introduced a new topic. Given that a new topic requires additional processing effort than sentences that are continuation of the same topic, it is probable that readers allocated additional resources to the updating of a discourse representation in topic-shifted condition, and thus resulted in the late positivity effect. Combined with the finding that discourse structure modulates the distribution of the N400 effect, our data provide evidence that discourse structure influences both the initial stage of meaning interpretation and the later stage of discourse updating during online discourse comprehension.
